# Suppressing Symptomless Nonhost Resistance of Barley to *Tobacco mosaic virus* by Short-Term Heat Stress—Role of Superoxide in Resistance

**DOI:** 10.3390/plants14172736

**Published:** 2025-09-02

**Authors:** Lóránt Király, Renáta Bacsó, Réka Albert, Ildikó Schwarczinger, Judit Kolozsváriné Nagy, András Künstler

**Affiliations:** 1 Department of Plant Pathophysiology, Plant Protection Institute, HUN-REN Centre for Agricultural Research, H-1116 Budapest, Hungary; 2 Institute of Plant Sciences and Environmental Protection, Faculty of Agriculture, University of Szeged, H-6800 Hódmezővásárhely, Hungary

**Keywords:** barley, nonhost resistance, *Tobacco mosaic virus*, heat stress, superoxide, reactive oxygen species, antioxidants, glutathione

## Abstract

Our previous research has demonstrated the role of optimal temperatures and reactive oxygen species (ROS) in maintaining symptomless nonhost resistance (NHR) of barley to powdery mildews. However, the exact functions of temperature and ROS in NHR of plants, including barley, to viral infections are not known. Although barley is a nonhost for *Tobacco mosaic virus* (TMV), this virus can replicate in barley leaves at temperatures of ca. 30 °C. Here we elucidated the influence of short-term heat stress pre-treatments (30 °C, 3 h; heat shock at 49 °C, 20 s) on the symptomless NHR of barley to TMV and the role of the ROS superoxide (O_2_^.−^) in maintaining NHR. Heat stress and antioxidant (superoxide dismutase and catalase, SOD + CAT) treatments resulted in 50–100% higher TMV levels, while combined heat shock and SOD + CAT application caused further increases in TMV and appearance of cell and tissue death resembling a hypersensitive response (HR). An early (from 2 h after inoculation) burst of O_2_^.−^ was essentially absent in TMV-infected barley exposed to short-term heat stress pre-treatments. Expression of barley genes regulating ROS (O_2_^.−^) metabolism (*HvRBOHF2*, *HvSOD1*) and cell death (*HvBI-1*) displayed an inverse correlation with TMV levels even at later time points (1–4 days after inoculation), implying a role in symptomless NHR, while increased levels of the antioxidant glutathione marked heat stress-induced suppression of NHR. We demonstrated that short-term heat stress and antioxidant treatments result in compromised NHR of barley to TMV, pointing to the role of optimal temperatures and ROS (O_2_^.−^) in symptomless NHR to virus infections.

## 1. Introduction

The first line of plant defense to pathogens involves recognition of pathogen-associated molecular patterns (PAMPs) by plant extracellular pathogen (pattern) recognition receptors (PRRs). This process confers basal (usually symptomless) resistance [1,2]. However, well-adapted pathogens secrete effectors to overcome recognition. Suppression of PAMP-triggered immunity (PTI) by pathogen effectors leads to effector-triggered susceptibility. In this case, a second line of plant defense may be induced, which is a cultivar and pathogen race-specific (“gene-for-gene”) resistance where plants recognize and inhibit pathogen effectors with the aid of resistance (R) proteins (effector-triggered immunity, ETI) [3,4,5]. Importantly, PTI and ETI are interdependent and mutually potentiate each other see [6] and references within).

Plant host resistance is very often governed by ETI and is usually associated with programmed cell and tissue death (PCD) localized at infection sites, termed the hypersensitive response (HR) [7,8,9]. This “gene-for-gene” resistance, however, can be regularly broken down by newly emerging pathogen races (pathogens evade detection by host *R* proteins by losing or mutating their corresponding effectors); therefore, it is not very durable [3,4]. In contrast, NHR is not cultivar and pathogen race-specific—it is a broad spectrum, durable resistance exhibited by all cultivars of a plant species against all races or isolates of a given pathogen [10]. Depending on the presence or absence of visual symptoms, NHR can be regarded as Type I (no visual symptoms, resembling PTI) or Type II (associated with an HR, similar to ETI) [11,12]. NHR is usually symptomless (Type I) when the phylogenetic distance between host and nonhost plants is significant (e.g., tobacco vs. barley). In this case, pathogen effectors fail to “find” most or all of their plant cellular targets, resulting in a very limited or totally absent pathogen replication (i.e., an intermediate or “true” Type I NHR, respectively). On the other hand, Type II (HR-associated) NHR may prevail when host and nonhost plant species are phylogenetically close (e.g., tobacco vs. tomato); therefore, pathogen effectors may recognize at least a few target proteins in the nonhost plant and induce a limited infection before the onset of resistance [13,14].

Most of the existing scientific literature describes plant NHR to fungal and bacterial pathogens. In fact, several key defense factors involved in host resistance (e.g., ROS, salicylic acid, pathogenesis-related proteins) also play a pivotal role in NHR of monocot and dicot plants to fungi and bacteria (for reviews see [11,12,15,16,17,18]. However, less data are available on possible mechanisms of NHR to plant viruses. The role of plant proteins inhibiting virus-encoded replication factors was shown in the NHR of tomato to *Tobacco mild green mosaic virus* (TMGMV) and *Pepper mild mottle virus* (PMMoV) [19], *Arabidopsis thaliana* to *Brome mosaic virus* (BMV) [20], and cucumber and Ethiopian mustard to potyviruses (*Plum pox virus*, PPV, and *Turnip mosaic virus*, TuMV, respectively) [21,22]. Furthermore, plant proteins participating in antiviral RNA-silencing contribute to NHR of *A. thaliana* to *Potato virus X* (PVX) [23]. Moreover, passive NHR to viral infections exists in plants expressing translation initiation factor isoforms not compatible with the invading virus [24]. In spite of the above-mentioned data, the general molecular and pathophysiological processes governing NHR of plants to virus infections still remain largely unexplored [25,26].

ROS accumulation in infected plants that resist pathogens has a dual role. Higher ROS concentrations (e.g., that of O_2_^.−^ or its dismutation product, hydrogen peroxide [H_2_O_2_]) may facilitate PCD of infected plant cells and the limitation or death of pathogens, including viruses, due to their toxicity [27]. However, at lower concentrations, ROS function as signaling agents activating plant defense responses to (viral) infections in healthy plant cells surrounding infection sites [28,29]. In fact, it is known that a relatively early (6 to 10 h after inoculation [HAI]) nicotinamide adenine dinucleotide phosphate (NADPH) oxidase-dependent O_2_^.−^ and H_2_O_2_ accumulation has a pivotal role in HR-associated resistance to plant viruses like TMV [30,31]. Down-regulation of NADPH oxidase by keeping plants at high (30 °C) temperatures or by transgenic means resulted in the elimination of HR-type TMV resistance and a pronounced decline of *in planta* O_2_^.−^ and H_2_O_2_ levels [32,33]. Importantly, we found that in TMV-susceptible tobacco the infiltration of inoculated leaves with an O_2_^.−^ -generating agent (riboflavin and methionine) at 2 HAI confers symptomless resistance to TMV [34]. In line with this, the role of a very early (1 to 6 HAI) accumulation of ROS (O_2_^.−^ and H_2_O_2_), in conferring rapid, symptomless extreme resistance (a form of ETI) to viruses such as PVX has also been shown [35,36].

High temperature-induced heat stress in plants causes serious problems in crop production. Barley (*Hordeum vulgare*) is an economically important cereal crop best adapted to temperate climates, although it is produced worldwide. Heat stress may decrease barley grain yield and quality, and, as in most land plants, usually compromises plant disease resistance [37,38]. For example, pre-exposures to long-term heat stress (35 °C for 1 to 5 days) negatively affect the resistance of barley to its powdery mildew pathogen (*Blumeria hordei,* Bh) in cv. Ingrid near-isogenic lines manifested as increased pathogen growth [39]. Furthermore, shorter heat exposures (36 °C for 30 min, 60 min, and 120 min) also cause enhanced susceptibility to Bh in both host-resistant and susceptible barley cultivars [40]. It is known that even a heat shock (HS, submerging plants to 48–49 °C water for 20–40 s) may cause a suppression of symptomless and HR-accompanied host resistance of barley to Bh [41,42].

Our previous research has demonstrated a rapid, early (1 day after inoculation, DAI) accumulation of O_2_^.−^ during symptomless NHR of barley to wheat powdery mildew (*B. graminis* f.sp. *tritici*, Bgt). Importantly, both O_2_^.−^ and NHR of barley to Bgt could be partially suppressed by HS (49 °C for 45 s) and antioxidant (SOD and CAT) treatments, pointing to the role of optimal temperatures and ROS in maintaining symptomless NHR of barley to powdery mildews [43]. Barley is also a nonhost for viruses like e.g., TMV, responding with symptomless NHR. However, it is known for decades that at daytime high temperatures of ca. 30 °C, TMV can replicate in barley leaves without causing any visible symptoms, suggesting at least a partial impairment of NHR [44,45].

Here we elucidated the influence of short-term heat stress pre-treatments (30 °C for 3 h or a HS at 49 °C for 20 s) on the NHR of barley to TMV and the role of an early ROS (O_2_^.−^) burst in maintaining this symptomless NHR at normal (20 °C) temperatures. An early (from 2 HAI) burst of O_2_^.−^ was essentially absent in TMV-infected barley exposed to short-term heat stress pre-treatments, concomitant with a compromised NHR. Expression of barley genes governing ROS (O_2_^.−^) metabolism and cell death regulation (*HvRBOHF2*, *HvSOD1*, *HvBI-1*) displayed an inverse correlation with TMV levels, implying a role in symptomless NHR of barley to TMV, while increased levels of the antioxidant glutathione marked heat-induced suppression of NHR. We demonstrate that short-term heat stress and antioxidant (SOD + CAT) treatments result in a compromised NHR of barley to TMV, pointing to the role of optimal temperatures and ROS (O_2_^.−^) in symptomless NHR to virus infections.

## 2. Results

### 2.1. Roles of Heat Exposure and Defense Gene Expression in Symptomless (Type I) Nonhost Resistance of Barley cv. Ingrid to TMV

Previously, it was shown that TMV can replicate in leaves of barley, a nonhost plant, without causing any visible symptoms. TMV replication and movement were dependent primarily on high daytime temperatures (ca. 30 °C) [44,45]. Based on these data, we tried to simulate conditions in Central European barley fields in May and June, when afternoon temperatures can easily reach ca. 30 °C and are sustained for several hours (see, e.g., [40]). Furthermore, in our earlier work, we have applied HS treatments (49 °C, 20–45 s, 30–120 min before pathogen inoculation) to suppress the symptomless (Type I) NHR of barley to wheat powdery mildew [43,46]. Therefore, in the present study, we wanted to clarify if similar heat exposures could also impair symptomless (Type I) nonhost resistance of barley to a virus, TMV?

**Figure 1:**
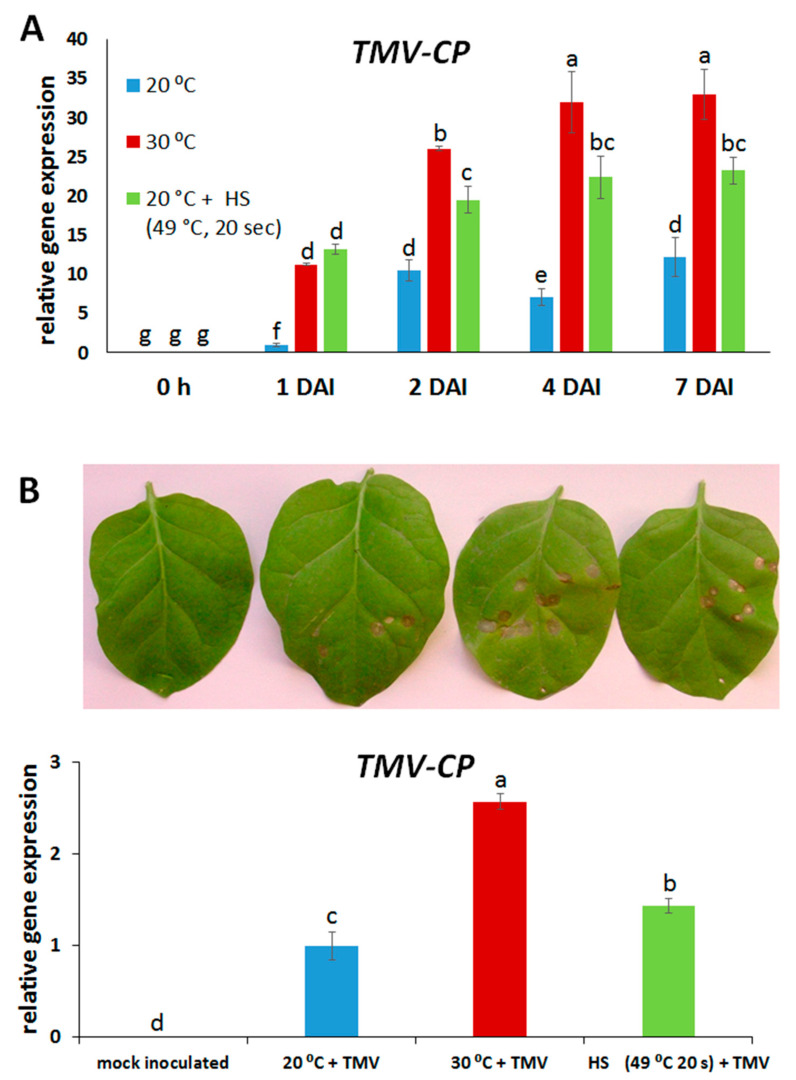
Accumulation of *Tobacco mosaic virus* (TMV) in barley cv. Ingrid, following pre-exposure to a heat shock (49 °C for 20 s, at 2 h before virus inoculation), or to 30 °C for 3 h or constantly kept at 20 °C, at 1, 2, 4, and 7 days after inoculation (DAI) (**A**). Expression of the TMV coat protein gene (*TMV-CP*) was assayed by real-time RT-qPCR and normalized to that of the reference gene *HvUbi*. Accumulation of TMV in leaves of *Nicotiana tabacum* cv. Xanthi NN NahG tobacco back inoculated with TMV-infected barley cv. Ingrid (**B**). Back inoculation was conducted 4 days after inoculation of cv. Ingrid barley with TMV (see (**A**)). Tobacco leaves photographed at 10 days after back inoculation. Photographs are from one representative experiment, which was repeated three times with similar results. Hypersensitive necrotic lesions (HR) indicate the presence of TMV (**upper panel**). Mock inoculated = back-inoculum without TMV; 20 °C = back-inoculum: TMV-infected barley constantly kept at 20 °C; 30 °C = back-inoculum: TMV-infected barley pre-exposed to 30 °C for 3 h before inoculation; Heat S (49 °C 20 s) = back-inoculum: TMV-infected barley pre-exposed to a heat shock (49 °C for 20 s) before inoculation. Expression of the TMV coat protein gene (*TMV-CP*) in back-inoculated *N. tabacum* cv. Xanthi NahG (**lower panel**) was assayed by real-time RT-qPCR and normalized to that of the reference gene *NtAct*. Graphs show the average of three experiments ± SD. Different letters indicate statistically significant differences at *p* ≤ 0.05.

Our results revealed that the short-term heat stress exposures applied (HS at 49 °C for 20 s, 2 h before inoculation; exposure to 30 °C for 3 h before inoculation) can significantly reduce barley (cv. Ingrid) NHR to TMV, as virus levels (assayed by real time reverse transcription quantitative PCR, real time RT-qPCR) were at least 50–100% higher in heat-stressed plants than during 20 °C at most investigated time points (1 to 7 days after inoculation/DAI/) (Figure 1A). Interestingly, a heat stress-compromised NHR was not associated with any visible symptoms in TMV-inoculated barley plants (Supplemental Figure S1).

To confirm the presence of infective TMV in the originally inoculated barley leaves, we have back-inoculated tobacco (*Nicotiana tabacum* cv. Xanthi NN NahG) with TMV-infected barley cv. Ingrid (exposed or not exposed to heat stress). Back inoculation resulted in the development of hypersensitive local necrotic lesions (HR) in tobacco (Figure 1B, upper panel), and lesion numbers correlated with TMV levels assayed in both back-inoculated tobacco and the original (inoculum source) barley leaves (compare Figure 1A and Figure 1B, lower panel).

**Figure 2:**
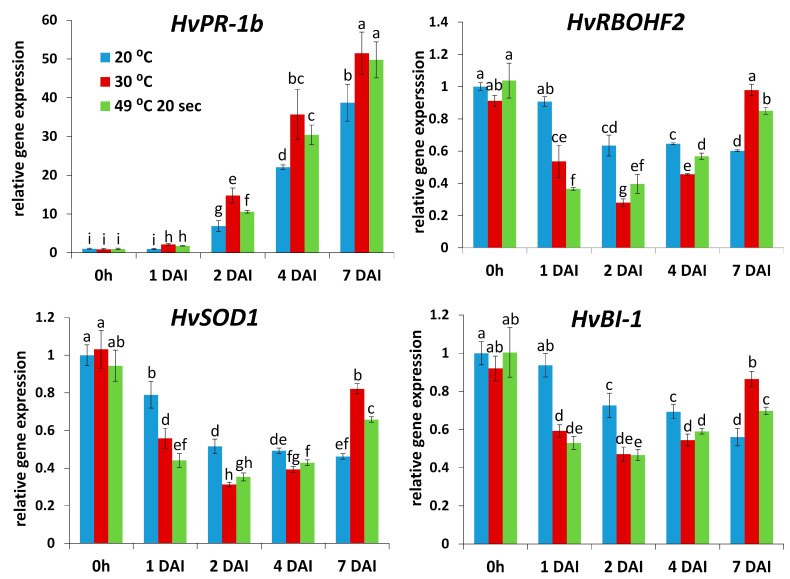
Expression of a pathogenesis-related (*HvPR-1b*), a NADPH oxidase (*HvRBOHF2*), a superoxide dismutase (*HvSOD1*), and a BAX-inhibitor gene (*HvBI-1*) in barley cv. Ingrid, following pre-exposure to a heat shock (49 °C for 20 s, at 2 h before virus inoculation), or to 30 °C for 3 h or constantly kept at 20 °C, at 1, 2, 4, and 7 days after *Tobacco mosaic virus* (TMV) inoculation (DAI). Gene expression was assayed by real-time RT-qPCR, normalized to that of the reference gene *HvUbi* and to mock-inoculated samples. Graphs show the average of three experiments ± SD. Different letters indicate statistically significant differences at *p* ≤ 0.05.

A heat stress-elicited breakdown of NHR to, e.g., viruses like TMV in barley could result from a partial collapse of the plant defense system. To test this hypothesis, we monitored transcription of several defense-related barley genes by real time RT-qPCR: a pathogenesis-related gene (*HvPR-1b*), a gene encoding NADPH oxidase (respiratory burst oxidase homolog) responsible for production of the ROS O_2_^.−^ and host disease resistance (*HvRBOHF2*), and *HvSOD1* (superoxide dismutase) and *HvBI-1* (BAX-inhibitor), genes encoding an antioxidant and a cell death regulator, respectively. Expression of *HvPR-1b* correlated with TMV levels, rather than NHR, indicating a function of this barley gene as a stress/susceptibility marker, a least in a late stage of this particular plant-pathogen interaction (1–7 DAI). On the other hand, expression of the remaining defense genes (*HvRBOHF2*, *HvSOD1*, *HvBI-1*) displayed an inverse correlation with TMV levels, i.e., an association with NHR, while gene expression sharply declined in heat-stressed barley where NHR was compromised (Figure 2). Importantly, these results imply that, at least to a certain extent, defense responses could have been retained in TMV-infected, nonhost-resistant barley (kept at 20 °C) even at a relatively late stage of pathogenesis (1 to 4 DAI).

**Figure 3:**
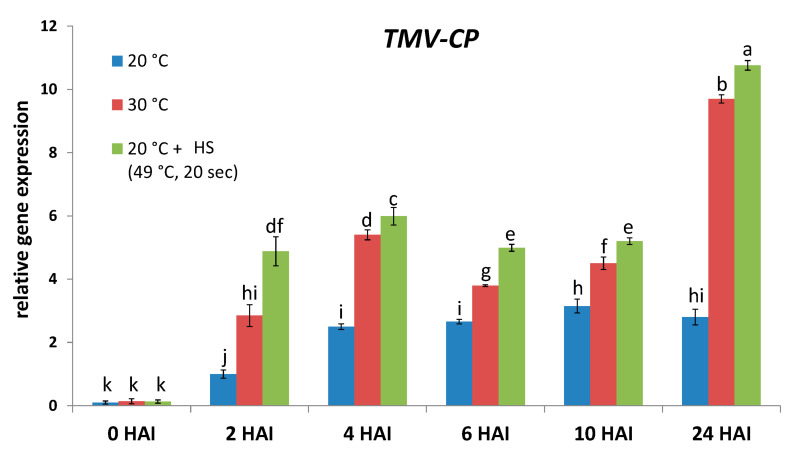
Accumulation of *Tobacco mosaic virus* (TMV) in barley cv. Ingrid, following pre-exposure to a heat shock (49 °C for 20 s, at 2 h before virus inoculation), or to 30 °C for 3 h or constantly kept at 20 °C, at 2, 4, 6, 10, and 24 h after inoculation (HAI). Expression of the TMV coat protein gene (*TMV-CP*) was assayed by real-time RT-qPCR and normalized to that of the reference gene *HvUbi*. Graph data points depict the average of three experiments ± SD. Different letters indicate statistically significant differences at *p* ≤ 0.05.

**Figure 4:**
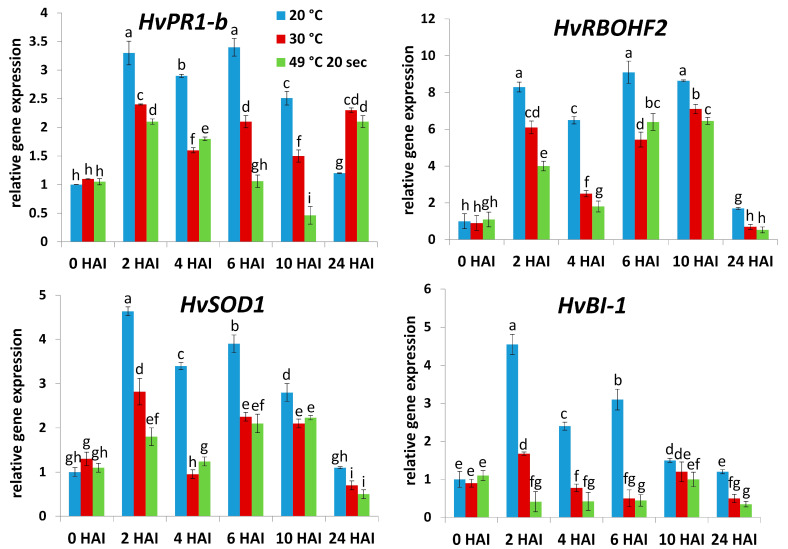
Expression of a pathogenesis-related (*HvPR-1b*), a NADPH oxidase (*HvRBOHF2*), a superoxide dismutase (*HvSOD1*), and a BAX-inhibitor gene (*HvBI-1*) in barley cv. Ingrid, following pre-exposure to a heat shock (49 °C for 20 s, at 2 h before virus inoculation), or to 30 °C for 3 h, or constantly kept at 20 °C, at 2, 4, 6, 10, and 24 h after *Tobacco mosaic virus* (TMV) inoculation (HAI). Gene expression was assayed by real-time RT-qPCR, normalized to that of the reference gene *HvUbi* and to mock-inoculated samples. Graphs show the average of three experiments ± SD. Different letters indicate statistically significant differences at *p* ≤ 0.05.

In order to see if the above-mentioned defense-related genes may contribute to the establishment of NHR to TMV in an early stage of pathogenesis, we assayed TMV levels and the expression of *HvPR-1b*, *HvRBOHF2*, *HvSOD1*, and *HvBI-1* in barley cv. Ingrid (TMV-infected and exposed or not exposed to heat stress) within the first few hours of pathogenesis (i.e., 1, 2, 3, 6, and 24 HAI). We found that the symptomless NHR of cv. Ingrid barley to TMV (at normal temperatures [20 °C] vs. heat-stress) was indeed evident as early as 2 to 6 HAI (assayed by RT-qPCR) (Figure 3). Importantly, expression of all investigated defense-related genes (*HvPR-1b*, *HvRBOHF2*, *HvSOD1*, and *HvBI-1*) correlated with NHR to TMV at these early time points (within 24 HAI), suggesting that these defense genes indeed take part in the establishment of NHR to TMV already within this early stage of pathogenesis (Figure 4).

### 2.2. Roles of ROS (O_2_^.−^) in Symptomless (Type I) Nonhost Resistance of Barley cv. Ingrid to TMV

To further monitor NHR-related defense processes in TMV-infected, heat-stressed barley, we conducted experiments to monitor the early accumulation of the ROS O_2_^.−^.

**Figure 5:**
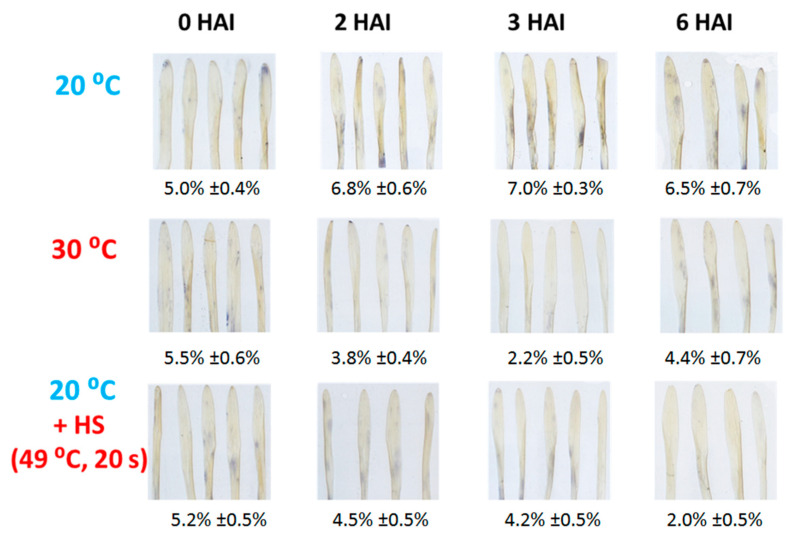
Absence of an early burst of superoxide (O_2_^.−^) in barley cv. Ingrid, following pre-exposure to a heat shock (49 °C for 20 s, at 2 h before virus inoculation), or to 30 °C for 3 h, or constantly kept at 20 °C, at 2, 4, and 6 h after *Tobacco mosaic virus* (TMV) inoculation (HAI). Superoxide production was detected by nitro blue tetrazolium chloride (NBT) staining. Photographs are from one representative experiment, which was repeated three times with similar results. Quantification of NBT-staining was conducted by ImageJ 1.54g (% leaf area stained with NBT). Values represent means ± SD.

We found that at 2, 3, and 6 HAI O_2_^.−^ accumulation was slightly but significantly lower in heat-stressed barley cv. Ingrid leaves infected with TMV, as compared to controls (kept at 20 °C), suggesting a role of O_2_^.−^ in NHR of barley to TMV (Figure 5). In order to confirm the finding that O_2_^.−^ levels indeed correlate with NHR of barley to TMV, we validated our NBT tissue staining results (O_2_^.−^ accumulation in TMV-infected barley cv. Ingrid leaves exposed or not exposed to heat stress) by quantitative image analysis using the Image J program “https://imagej.net/ij/ (accessed on 30 August 2025)”. This revealed that in heat-stressed barley cv. Ingrid leaves infected with TMV O_2_^. −^ levels were only ca. 30–50% of those in control plants (kept at 20 °C) (Figure 5).

**Figure 6:**
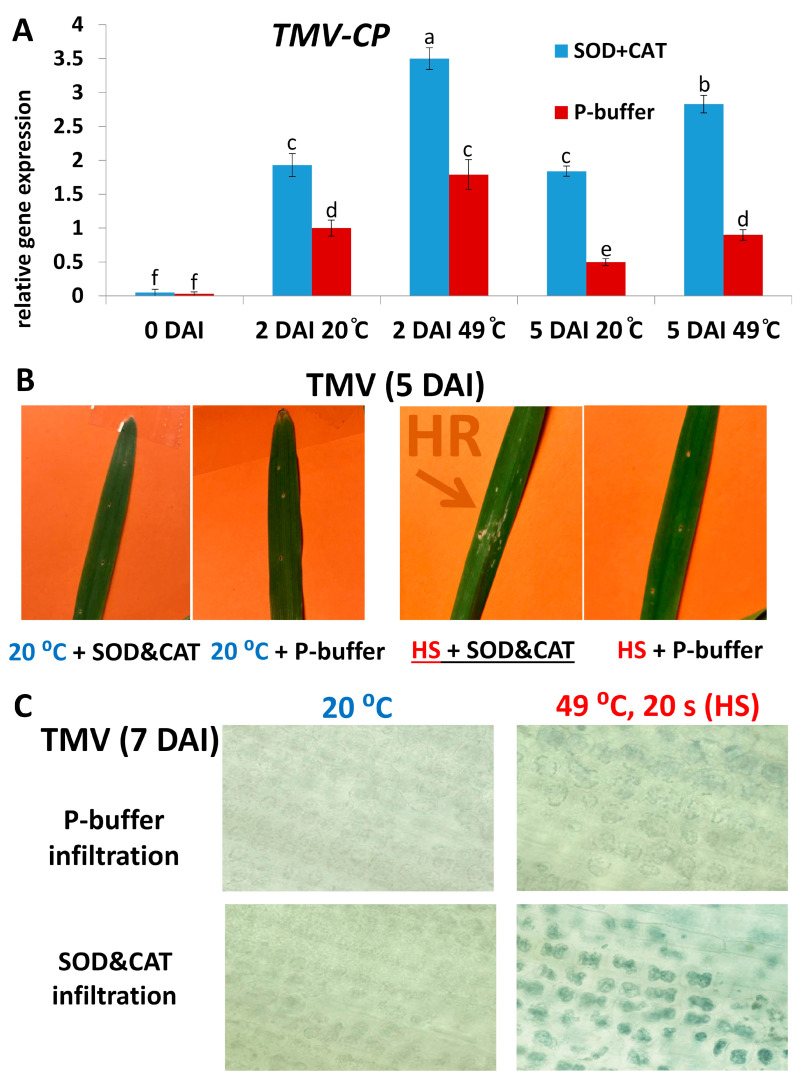
Accumulation of *Tobacco mosaic virus* (TMV) in barley cv. Ingrid, in response to a heat shock pre-treatment (49 °C = 49 °C heat shock for 20 s, at 2 h before virus inoculation; 20 °C = control treatment) and leaf infiltration of antioxidants (blue columns = SOD, 2500 U/mL + CAT, 5000 U/mL; red columns = control infiltration: 10 mM K-phosphate buffer, pH 7.5), at 2 and 5 days after inoculation (DAI) (**A**). Expression of the TMV coat protein gene (*TMV-CP*) was assayed by real-time RT-qPCR and normalized to that of the reference gene *HvUbi*. Graph data points depict the average of three experiments ± SD. Different letters indicate statistically significant differences at *p* ≤ 0.05. Combined application of heat shock (HS) pre-treatment (49 °C, 20 s) and leaf infiltration of antioxidants (SOD&CAT) in cv. Ingrid barley inoculated with TMV may cause the appearance of visible necrotic lesions resembling a hypersensitive response (HR) and indicating programmed cell death (PCD) (**B**). Heat shock (HS) pre-treatment (49 °C, 20 s), especially when combined with leaf infiltration of antioxidants (SOD and CAT) in cv. Ingrid barley inoculated with TMV may cause death of mesophyll cells/chloroplasts even in the absence of a visible HR (**C**). Barley cell death as visualized by Evans Blue staining. In case of (**B**,**C**), photographs are from one representative experiment, which was repeated three times with similar results.

If the ROS O_2_^.−^ indeed contributes to the NHR of barley to TMV, then the inhibition of O_2_^.−^ accumulation by, e.g., antioxidant treatments should at least partially inhibit NHR (i.e., increase TMV levels). Infiltration of the antioxidant enzymes SOD (2500 U/mL) and CAT (5000 U/mL) into barley (cv. Ingrid) leaves immediately after inoculation has significantly increased TMV levels as compared to control (buffer-infiltrated) plants. This increase in TMV titers was comparable to that caused by HS (49 °C, 20 s) pre-treatments: at 2 and 5 DAI, both antioxidant and HS treatments alone resulted in at least 50–100% higher TMV levels (Figure 6A). Remarkably, simultaneous application of HS and SOD + CAT infiltration caused an even higher (almost an order of magnitude) increase in TMV titers (Figure 6A) and often the appearance of visible, hypersensitive (HR) type local necrotic symptoms, a possible indicator of PCD and a compromised symptomless (Type I) nonhost virus resistance (Figure 6B). Interestingly, we found that even in those cases where a simultaneous application of HS (49 °C, 20 s) and SOD + CAT infiltration did not result in visible, HR-type local necrotic symptoms, death of individual mesophyll cells and their chloroplasts could be detected in TMV-inoculated barley leaves. In fact, sporadic mesophyll cell and chloroplast death occurred even when leaves were exposed only to HS (Figure 6C).

**Figure 7:**
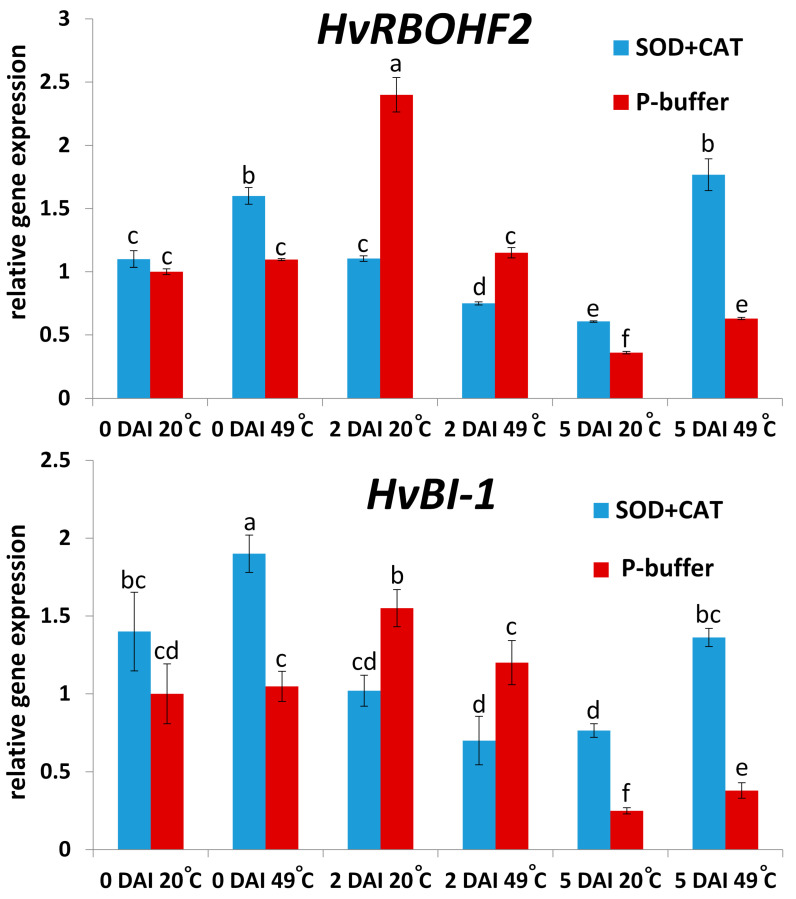
Expression of defense-related genes responsible for ROS (superoxide) production and resistance (*HvRBOHF2*) and PCD regulation (*HvBI-1*) in cv. Ingrid barley, 2 and 5 days after *Tobacco mosaic virus* (TMV) inoculation (DAI), in response to a heat shock pre-treatment (49 °C = 49 °C heat shock for 20 s, at 2 h before virus inoculation; 20 °C = control treatment) and leaf infiltration of antioxidants (blue columns = SOD, 2500 U/mL + CAT, 5000 U/mL; red columns = control infiltration: 10 mM K-phosphate buffer, pH 7.5). Gene expression was assayed by real-time RT-qPCR and normalized to that of the reference gene *HvUbi* and to mock-inoculated samples. Graphs show the average of three experiments ± SD. Different letters indicate statistically significant differences at *p* ≤ 0.05.

Our results also revealed that at 2 DAI, symptomless NHR to TMV (at 20 °C, without SOD + CAT infiltration; see Figure 6A) was correlated with enhanced expression of barley defense genes responsible for O_2_^.−^ production/resistance (*HvRBOHF2*), and programmed cell death (PCD) regulation (*HvBI-1*) (Figure 7). However, at a later time point (5 DAI) *HvRBOHF2* and *HvBI-1* induction was rather associated with a compromised NHR to TMV (exposure to 49 °C HS and SOD + CAT infiltration; see Figure 6A) (compare Figure 6A and Figure 7), i.e., at later stages of pathogenesis these genes seem to function as stress markers that correlate with susceptibility, rather than resistance (Figure 7).

### 2.3. Glutathione May Take Part in Suppressing Symptomless (Type I) Nonhost Resistance of Barley cv. Ingrid to TMV at Later Stages of Infection

**Figure 8:**
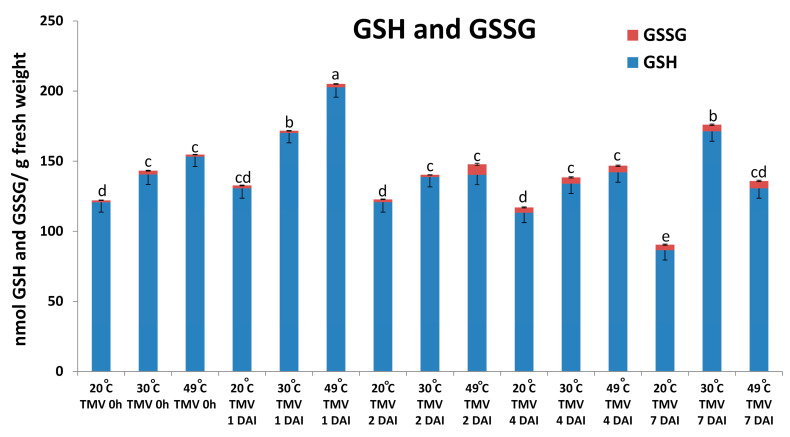
Levels of reduced and oxidized glutathione (GSH and GSSG) in barley cv. Ingrid, following pre-exposure to a heat shock (49 °C = 49 °C for 20 s, at 2 h before virus inoculation), or to 30 °C for 3 h (30 °C) or constantly kept at 20 °C (20 °C), at 1, 2, 4, and 7 days after *Tobacco mosaic virus* (TMV) inoculation (DAI). Glutathione was assayed by HPLC separation and electrospray ionization mass spectrometric analyses (HPLC-ESI/MS). Graph data points depict the average of three experiments ± SD. Different letters indicate statistically significant differences in levels of total glutathione (GSH and GSSG) at *p* ≤ 0.05.

Our results demonstrated that the infiltration of antioxidant enzymes (SOD + CAT) can significantly suppress the NHR of barley cv. Ingrid to TMV (see Figure 6). We wanted to clarify if a non-enzymatic antioxidant like glutathione may also take part in suppressing the NHR of barley to TMV (i.e., in maintaining partial susceptibility)? Levels of reduced and oxidized glutathione (GSH and GSSG) were assayed. In fact, our experiments revealed that glutathione levels (essentially the reduced form, GSH) indeed significantly increase during heat stress-induced suppression of symptomless (Type I) NHR of barley cv. Ingrid to TMV at later stages of virus infection (1 to 7 DAI) (Figure 8).

## 3. Discussion

It is known for several decades that TMV can replicate in leaves of barley, a nonhost plant, without causing any visible symptoms. These early studies showed that TMV replication and movement were dependent primarily on high daytime temperatures (ca. 30 °C) [44,45]. Our results revealed that even the short-term heat stress exposures applied in the present study (HS at 49 °C for 20 s, 2 h before inoculation; exposure to 30 °C for 3 h before inoculation) can significantly reduce barley nonhost resistance (NHR) to TMV, as virus levels were at least 50–100% higher in heat-stressed plants than during 20 °C, without eliciting any obvious symptoms. Importantly, back inoculation of tobacco (cv. Xanthi NahG) with TMV-infected barley (exposed or not exposed to heat stress) resulted in the development of the expected hypersensitive local necrotic lesions (HR) in tobacco, and lesion numbers correlated with TMV levels both in back-inoculated tobacco and the original (inoculum source) barley leaves. In fact, virus replication was detectable (assayed by RT-qPCR) even in samples derived from TMV-inoculated barley not exposed to heat stress pre-treatments (kept at 20 °C). This implies that the NHR of barley cv. Ingrid to TMV might be regarded as an “apparent” or intermediate NHR where the nonhost plant species allows a very limited multiplication of the invading virus in single or a few inoculated cells, as opposed to “true NHR” (i.e., a complete absence of virus replication) [21,25,26]. In this context, the 50–100% higher TMV titers in heat-stressed barley may represent intermediate (limited) susceptibility, as part of a continuum between nonhost and host plant responses to pathogen infections. Besides heat stress, several other environmental and physiological factors (humidity, circadian clock, photoperiod, developmental stage, tissue context) may confer a shift from, e.g., intermediate NHR to intermediate susceptibility [18].

Our results demonstrating that short-term heat stress pre-treatments (including HS) significantly reduce barley symptomless (Type I) NHR to TMV are in line with earlier reports. These show the suppression of symptomless and HR-accompanied host resistance of barley to its powdery mildew pathogen (Bh) [41,42] and symptomless (Type I) NHR of barley to wheat powdery mildew (Bgt) [43,46] by similar HS pre-treatments (48–49 °C, 20–45 s, 30–120 min before inoculation).

A heat stress-elicited deterioration of NHR to, e.g., viruses like TMV in barley could result from a partial collapse of the plant defense system. We found that enhanced expression of a pathogenesis-related barley gene (*HvPR-1b*) correlated with symptomless NHR to TMV only in early stages of pathogenesis (within 24 HAI), while at later time points (1–7 DAI) *HvPR-1b* expression associated with heat stress-induced higher TMV levels, rather than NHR, indicating a function of this barley gene as a stress/susceptibility marker in later stages of viral pathogenesis. This trend is similar to previously reported data showing that in tobacco displaying symptomless extreme resistance to PVX the induction of *PR-1* genes occur in initial stages of viral pathogenesis (1–24 HAI) [36,47], while elevated *PR-1* transcription in advanced stages of, e.g., TMV infection is clearly a marker of host stress and/or a failed resistance response [48,49].

Importantly, however, barley defense-related genes regulating ROS (O_2_^.−^) metabolism (*HvRBOHF2*, *HvSOD1*) and cell death (*HvBI-1*) displayed an inverse correlation with TMV levels even at later time points (1–4 DAI), suggesting a role of ROS (O_2_^.−^) and cell death regulation in the establishment of symptomless NHR of barley to TMV. Indeed, we found that an early (from 2 to 6 HAI) burst of O_2_^.−^ was essentially absent in TMV-infected barley exposed to short-term heat stress pre-treatments, concomitant with a compromised NHR. Interestingly, the highest transcript levels of *HvRBOHF2* (encoding a NADPH oxidase [respiratory burst oxidase homolog] responsible for O_2_^.−^ production), *HvSOD1* (encoding a O_2_^.−^ dismutase) and *HvBI-1* (encoding an inhibitor of PCD) occurred in barley displaying symptomless NHR to TMV at early time points, mirroring O_2_^.−^ accumulation (2–6 HAI). This corroborates previous observations in barley showing symptomless NHR to wheat powdery mildew (Bgt) and in PVX-infected, extreme resistant tobacco [36,43]. Furthermore, it is known that a tight regulation of NADPH oxidase-driven O_2_^.−^ production is a central component of virus (e.g., TMV) resistance [30,31,32,33]. Also, *BI-1* (BAX inhibitor-1) genes are not only PCD-regulators during plant stress responses [50] but may contribute to resistance to viral, bacterial, and fungal pathogens in wheat and *Nicotiana benthamiana* [51,52,53]. Interestingly, however, we found that at late time points (5 and 7 DAI), *HvRBOHF2* and *HvBI-1* induction were rather associated with a compromised NHR to TMV. This implies that at later stages of pathogenesis, these genes may function as susceptibility and stress markers, a phenomenon similar to that observed with plant *PR* genes during pathogen (e.g., virus) attacks (see above) [48,49]. In barley displaying symptomless NHR vs. HR-type host resistance to wheat and barley powdery mildew, respectively, an early (1 DAI), transient induction of *RBOH*-encoded NADPH oxidase activity and elevated *HvBI-1* expression is correlated with a fast, symptomless NHR, while a later (3 DAI) induction of these factors marks the slower development of HR-type host resistance [43]. Also, a transient, elevated early (within 1 DAI) expression of *NtBI-1* and NADPH oxidase activity in tobacco marks the development of fast, symptomless extreme resistance to PVX, while the same changes occur much later (2 to 5 DAI) during a slower HR to TMV infection, reflecting opposing functions of these biochemical changes in rapid defense vs. a less effective resistance (susceptibility) and stress [36].

If ROS (O_2_^.−^) indeed contributes to the symptomless NHR of barley to TMV, then the inhibition of O_2_^.−^ accumulation by, e.g., antioxidant treatments should at least partially inhibit NHR (i.e., increase TMV levels). Infiltration of antioxidant enzymes (SOD + CAT) resulted in significantly (50–100%) increased TMV levels, an effect comparable to that caused by HS (49 °C, 20 s) pre-treatments. Remarkably, simultaneous application of HS and SOD + CAT infiltration caused an even higher (almost an order of magnitude) increase in TMV titers and often the appearance of visible, hypersensitive (HR) type local necrotic symptoms, possible indicators of PCD, and a compromised symptomless (Type I) NHR of barley to TMV. A similar phenomenon occurs during a partial suppression of symptomless NHR of barley to wheat powdery mildew (Bgt) by a combined application of HS pre-treatments (49 °C, 45 s, 30 min before inoculation) and SOD + CAT infiltration [43]. Previously, we have also shown a partial suppression of symptomless extreme resistance to PVX by the same antioxidant (SOD + CAT) infiltrations, in parallel with the appearance of localized leaf necrosis resembling HR [36]. In fact, a TMV-infected transgenic tobacco overexpressing catalase (CAT) displays significantly larger HR-type necrotic lesions, indicating enhanced susceptibility (i.e., enhanced virus replication and/or movement) [54]. A link between heat stress, ROS suppression (antioxidant induction), and compromised virus resistance is demonstrated in our earlier work, showing that in tobacco kept at 30 °C, a suppression of HR-type resistance to TMV is associated with the down-regulation of ROS and induction of the antioxidant enzyme dehydroascorbate reductase [33]. Although in the present study we have demonstrated that short-term heat stress and antioxidant treatments result in compromised NHR of barley to TMV, the role of heat stress and ROS suppression in compromising NHR to viruses other than TMV is not known so far.

Interestingly, we found that even when a simultaneous application of HS and antioxidants did not result in visible, HR-type local necrotic symptoms in TMV-inoculated barley leaves, the death of individual mesophyll cells and their chloroplasts could be detected. This phenomenon might represent “micro-HR”—part of a continuum of temporal and spatial responses to, e.g., virus infections that range from the fastest symptomless (extreme) resistance through single-cell (micro) and the slower macroscopic HR, while a partial or total absence of defense responses, including ROS (O_2_^.−^) accumulation, may lead to susceptibility [28,55,56]. In addition, the possible death or disintegration of chloroplasts in these apparently dead mesophyll cells could mark a compromised NHR to TMV, indicating perturbations in ROS (O_2_^.−^) signaling from the chloroplast to other cell compartments that are required for efficient defense to pathogens (see, e.g., [57]. This is supported by a recent finding that the presence of mesophyll cells with moderately oxidized (but live) chloroplasts in *Potato virus Y* (PVY) infected potato leaves is associated with proper resistance signaling [58].

We have demonstrated that infiltration of antioxidant enzymes like SOD and CAT can initiate suppression of barley NHR to TMV, likely by causing perturbations in defense-related ROS (O_2_^.−^) signaling. However, our experiments also suggest that a non-enzymatic antioxidant like glutathione may, in fact, take part in maintaining the suppressed NHR of barley to TMV (i.e., maintaining partial susceptibility). Levels of glutathione (essentially the reduced form, GSH) indeed significantly increase at later stages of virus infection (1 to 7 DAI), concomitant with heat stress-induced suppression of symptomless (Type I) NHR of barley to TMV and a plausible plant response (antioxidant induction) to the enhanced cell death observed during NHR suppression. At present, we do not know whether glutathione can actively participate as a signaling agent in suppressing NHR to TMV. Glutathione has two fundamental roles in infected plants. As an antioxidant, glutathione may suppress host cell and tissue death during, e.g., an HR-type resistance elicited by viruses like TMV [49,59,60]. In powdery mildew (Bh) infected barley, glutathione contents significantly increase in susceptible plants, but only at late stages (7 DAI) of pathogen invasion, a likely requirement for a compatible infection without cell and tissue death [61]. However, in the initial stages of pathogenesis, glutathione is also a signaling agent with a role in disease resistance—elevated *in planta* glutathione levels during incompatible and compatible plant-virus interactions confer suppression of both symptoms and virus titers [49,62,63,64,65]. Although barley displaying NHR to TMV glutathione levels did not change significantly at later stages of infection (1 to 7 DAI), it cannot be ruled out that higher-than-normal levels of glutathione may contribute to TMV-elicited NHR at an earlier stage of pathogenesis. In fact, De et al. [66] showed that in PVX-infected *N. benthamiana*, inhibition of glutathione accumulation by silencing a key biosynthesis gene (*GSH1*) increases virus replication.

Taken together, we have demonstrated that short-term heat stress and antioxidant treatments result in compromised NHR of barley to TMV, pointing to the role of optimal temperatures and ROS in symptomless NHR to virus infections. In facing the challenge of global climate change, our results may contribute to breeding crops for tolerance to combined stresses like heat and pathogen infections, which should be a primary task in the near future.

## 4. Materials and Methods

### 4.1. Plants and Pathogen, Virus Inoculation

Barley (*Hordeum vulgare*) cv. Ingrid was used in our experiments involving virus infections and short-term heat stress pre-treatments. Barley plants grown from ca. 30 seeds sown into two pots per treatment were kept in controlled environmental chambers (20 °C, 60% relative humidity, 16 h light/8 h dark photoperiod with a light intensity of 100 µmol m^−2^ s^−1^). For back inoculation, the SA-deficient *Nicotiana tabacum* cv. Xanthi NN NahG tobacco line [67], while for virus maintenance, *N. tabacum* cv. Samsun nn was used. Tobacco plants were grown in a greenhouse with standard parameters (temperatures between 20–23 °C, approximately 16 h of daylight, and daily watering).

The common (U1) strain of *Tobacco mosaic virus* (TMV) was maintained on the susceptible host *N. tabacum* cv. Samsun nn. Systemically infected young upper leaves with strong mosaic symptoms were used for further virus inoculations. Upper leaves systemically infected with TMV were homogenized in tap water (1 g of leaf in 10 mL of water) with silicon carbide (carborundum) powder as an abrasive and used to inoculate healthy leaves of 7–8-day-old barley by mechanical inoculation. Back inoculation was carried out using 1 g of leaves from barley plants (4 days after TMV inoculation) to infect *N. tabacum* cv. Xanthi NN NahG.

### 4.2. Short-Term Heat Stress Pre-Treatments of Barley

The following short-term heat stress pre-treatments were applied in 7–8-day-old barley inoculated with TMV: 1/exposure to 30 °C for 3 h before inoculation in controlled environmental chambers (60% relative humidity, 100 µmol m^−2^ s^−1^ light intensity); 2/heat shock (HS) by using a previously established protocol [41,42,43]. The first leaves of 7–8-day-old intact barley plants were immersed in a 49 °C water bath for 20 s, 2 h before TMV inoculation. Control plants were constantly kept at 20 °C in environmental chambers (see above).

### 4.3. Virus Accumulation and Gene Expression Analyses

Virus (TMV) accumulation and expression of defense-related genes were monitored in inoculated barley leaves and TMV accumulation in back-inoculated tobacco (cv. Xanthi NN NahG) by reverse transcription (RT) followed by quantitative (real-time) PCR (qPCR) essentially as described [36,68].

Total RNA (plant and virus) extraction was done from 100 mg of fresh leaves/sample homogenized in liquid nitrogen with the aid of a Plant Total RNA Extraction Miniprep System kit according to the instructions of the manufacturer (Viogene BioTek Corp., New Taipei City, Taiwan). RNA quantity and quality were assessed by a MaestroNano Spectrophotometer (Maestrogen, Hsinchu City, Taiwan). Subsequent RT from 1 μg of total RNA/sample was conducted with a RevertAid H– cDNA Synthesis Kit (Thermo Fisher Scientific, Waltham, MA, USA), by using both *TMV-CP* gene reverse and oligo-dT primers. qPCR was carried out for assaying TMV levels in barley and tobacco and expression of defense-related genes (*HvPR-1b*, *HvRBOHF2*, *HvSOD1*, and *HvBI-1*) in barley. qPCRs were run in a CFX-96 real-time thermocycler (Bio-Rad Laboratories, Inc., Hercules, CA, USA) with the aid of the 2× SYBR FAST ReadyMix Reagent (KAPA Biosystems, Wilmington, MA, USA). All reactions were performed with three technical replicates per biological sample. Primer efficiencies were between 100 and 104% for the genes tested; therefore, gene expression changes were calculated by using the 2^−∆∆CT^ method [69].

In tobacco, *TMV-CP* gene expression was normalized to an actin gene (*NtAct*) as a reference, while in barley, expression of *TMV-CP* and defense-related genes was normalized to an ubiquitin reference gene (*HvUbi*). Expression of defense-related barley genes in TMV-inoculated plants was also normalized to that of mock-inoculated controls. The suitability of *NtAct* as a reference gene in response to TMV inoculation has been confirmed earlier (see, e.g., [36]), while that of the *HvUbi* reference gene was tested by analysis of cycle threshold variation: significant changes in cycle threshold values (mean ± SD) were not observed during TMV infection. Previous research has also demonstrated that *HvUbi* is a reliable reference gene for monitoring gene expression in barley exposed to heat stress and SOD + CAT treatments and various infections [39,43,68,70].

Oligonucleotide primers used in RT-qPCR are described in Table 1. All oligonucleotide primers, except those for the genes *HvPR-1b* and *HvUbi* [71], were designed by us with the aid of the Primer Premier 5 program (Premier Biosoft International, San Francisco, CA, USA).

### 4.4. Antioxidant Treatments and Detection of Superoxide (O_2_^.−^) and Cell Death in Barley Leaves

Simultaneous infiltration of the antioxidant enzymes superoxide dismutase and catalase (SOD and CAT, 2500 and 5000 units mL^−1^, equivalent to 0.8 and 1.4 mg protein mL^−1^, respectively) (Sigma Aldrich/Merck Group, Rahway, NJ, USA) into barley leaves was conducted immediately after inoculation, as described earlier [43,72]. Infiltration of SOD and CAT was carried out with a syringe and needle. This implies that SOD and CAT enter the intercellular space and neutralize primarily the ROS produced by pathogen (virus) activated, plant plasma membrane-bound NADPH oxidases. Control infiltrations were carried out with 10 mM K-phosphate buffer (pH 7.5).

Accumulation of the ROS superoxide (O_2_^.−^) in barley leaves inoculated with TMV with or without heat stress pre-treatments was detected by histochemical staining with 0.1% (*w*/*v*) nitro blue tetrazolium chloride (NBT) (Sigma Aldrich/Merck Group, Rahway, NJ, USA) by vacuum infiltration according to the procedure of Ádám et al. [73]. Infiltrated leaf samples were incubated under daylight for 20 min, then cleared in a solution containing 0.15% (*w*/*v*) trichloroacetic acid in ethanol: chloroform 4:1 (*v*/*v*) and stored in 50% (*v*/*v*) glycerol until photography [74]. O_2_^.−^ accumulation (percentage of NBT-stained area per leaf) was quantified by using the ImageJ program “https://imagej.net/ij/ (accessed on 30 August 2025)”.

Cell death in leaves of TMV-inoculated barley plants exposed or not exposed to HS and SOD + CAT treatments was monitored by Evans blue tissue staining essentially as described earlier by Pogány et al. [75]. Leaves were vacuum infiltrated with 1.3 mM Evans blue (Fluka Chemie GmbH, Buchs, Switzerland) solution (0.125 g of Evans blue dissolved in 100 mL of distilled water). After 15 min of incubation in the dark, leaves were washed with distilled water and transferred to the same clearing solution used for NBT staining and then stored in 50% (*v*/*v*) glycerol (see above). For microscopic imaging of cell death in barley leaves, an Olympus BX51 light microscope (Olympus Corporation, Tokyo, Japan) was used.

### 4.5. Glutathione Assays

Determination of reduced (GSH) and oxidized (GSSG) glutathione was conducted as described by us earlier [49]. In brief, barley leaf tissue samples (500 mg) were homogenized in liquid nitrogen and resuspended in four volumes of cold (4 °C) extraction solution containing 5% (*w*/*v*) stabilized metaphosporic acid (Acros Organics, Geel, Belgium) and 1 mM EDTA in 0.1% formic acid, supplemented with 1% (*w*/*v*) polyvinyl-polypyrrolidone (PVP) just before use. The suspension was centrifuged at 14,000× *g* for 15 min at 4 °C, and supernatants filtered through 0.45 μm Cromafil polyamide membrane filters (Macherey-Nagel, Düren, Germany). After HPLC separation and electrospray ionization (HPLC-ESI), mass spectrometric analyses (MS) were performed with an HPLC MS system (Shimadzu Inc., Columbia, MD, USA) as described in detail by Künstler et al. [49]. Unlabeled, analytical grade GSH and GSSG (Sigma Aldrich/Merck Group, Rahway, NJ, USA) were used as external standards for quantification of GSH and GSSG levels in barley leaves.

### 4.6. Statistical Analysis

Three independent biological experiments were conducted in each case, with three replicates per treatment. Each biological sample contained at least 6–10 leaves collected from different individual plants. Statistical analyses were carried out with the aid of the ANOVA method followed by the Tukey post hoc test, employing the Statistica 13 software (TIBCO Software, Palo Alto, CA, USA). Differences at *p* ≤ 0.05 were considered statistically significant.

## Figures and Tables

**Table 1:** Oligonucleotide primers used in qPCR.

Accession Number	Gene	Sequence 5′-3′		Amplicon Length	Primer Efficiency
AF165190 and AJ429078	*Tobacco mosaic virus* *coat protein* (*TMV-CP*)	Fw	CTTGTCATCAGCGTGGGC	165 bp	101%
		Rev	AAGTCACTGTCAGGGAAC		
X69885	*Tobacco actin* (*NtAct*)	Fw	CGGAATCCACGAGACTACATAC	230 bp	100%
		Rev	GGGAAGCCAAGATAGAGC		
M60175	*Barley ubiquitin* (*HvUbi*)	Fw	ACCCTCGCCGACTACAACAT	240 bp	102%
		Rev	CAGTAGTGGCGGTCGAAGTG		
KU179438	*Barley cytosolic copper zinc superoxide dismutase* (*HvSOD1*)	Fw	TCAAGGGCACCATCTTCTTC	214 bp	102%
		Rev	TTTCCGAGGTCACCAGCAT		
AJ290421	*Barley BAX inhibitor-1* (*HvBI-1*)	Fw	ATGTTCTCGGTGCCAGTCT	409 bp	101%
		Rev	GGGCGTGCTTGATGTAGTC		
X74940	*Barley pathogenesis-related-1 b* (*HvPR1-b*)	Fw	GGACTACGACTACGGCTCCA	150 bp	104%
		Rev	GGCTCGTAGTTGCAGGTGAT		
EU566856	*Barley respiratory burst oxidase homolog F2* (*HvRBOHF2*)	Fw	TGCTCGGTCAGCACTC	175 bp	104%
		Rev	TCCGCAATAGAACACTCC		

## Data Availability

Data are contained within the article and Supplementary Materials.

## Supplementary Materials

The following supporting information can be downloaded at: https://www.mdpi.com/article/10.3390/plants14172736/s1, Figure S1. Heat stress-compromised nonhost resistance is not associated with any visible symptoms in barley cv. Ingrid plants, at 7 days after Tobacco mosaic virus (TMV) inoculation (DAI). Before virus inoculation, barley plants were either constantly kept at 20 °C (20 °C) or pre-exposed to 30 °C for 3 h (30 °C) or to a heat shock (49 °C = 49 °C for 20 s, at 2 h before virus inoculation).

## References

[1] Zipfel C. (2009). Early molecular events in PAMP-triggered immunity. Curr. Opin. Plant Biol..

[2] Lacombe S., Rougon-Cardoso A., Sherwood E., Peeters N., Dahlbeck D., van Esse H.P., Smoker M., Rallapalli G., Thomma B.P.H.J., Staskawicz B. (2010). Interfamily transfer of a plant pattern-recognition receptor confers broad-spectrum bacterial resistance. Nat. Biotechnol..

[3] Flor H.H. (1971). Current status of the gene for gene concept. Annu. Rev. Phytopathol..

[4] Dangl J.L., Horvath D.M., Staskawicz B.J. (2013). Pivoting the plant immune system from dissection to deployment. Science.

[5] Bentham A.R., De la Concepcion J.C., Mukhi N., Zdrzałek R., Draeger M., Gorenkin D., Hughes R.K., Banfield M.J. (2020). A molecular roadmap to the plant immune system. J. Biol. Chem..

[6] Pruitt R.N., Gust A.A., Nürnberger T. (2021). Plant immunity unified. Nat. Plants.

[7] Goodman R.N., Novacky A.J. (1994). The Hypersensitive Reaction in Plants to Pathogens.

[8] Künstler A., Bacsó R., Gullner G., Hafez Y.M., Király L. (2016). Staying alive—Is cell death dispensable for plant disease resistance during the hypersensitive response?. Physiol. Mol. Plant Pathol..

[9] Balint-Kurti P. (2019). The plant hypersensitive response: Concepts, control and consequences. Mol. Plant Pathol..

[10] Heath M.C. (2000). Nonhost resistance and nonspecific plant defenses. Curr. Opin. Plant Biol..

[11] Mysore K.S., Ryu C.-M. (2004). Nonhost resistance: How much do we know?. Trends Plant Sci..

[12] Senthil-Kumar M., Mysore K.S. (2013). Nonhost resistance against bacterial pathogens: Retrospectives and prospects. Annu. Rev. Phytopathol..

[13] Schulze-Lefert P., Panstruga R. (2011). A molecular evolutionary concept connecting nonhost resistance, pathogen host range, and pathogen speciation. Trends Plant Sci..

[14] McLellan H., Harvey S.E., Steinbrenner J., Armstrong M.R., He Q., Clewes R., Pritchard L., Wang W., Wang S., Nussbaumer T. (2022). Exploiting breakdown in nonhost effector-target interactions to boost host disease resistance. Proc. Natl. Acad. Sci. USA.

[15] Niks R.E., Marcel T.C. (2009). Nonhost and basal resistance: How to explain specificity?. New Phytol..

[16] Gill U.S., Lee S., Mysore K.S. (2015). Host versus nonhost resistance: Distinct wars with similar arsenals. Phytopathology.

[17] Lee H.A., Lee H.Y., Seo E., Lee J., Kim S.B., Oh S., Choi E., Choi E., Lee S.E., Choi D. (2017). Current understandings of plant nonhost resistance. Mol. Plant-Microbe Interact..

[18] Panstruga R., Moscou M.J. (2020). What is the molecular basis of nonhost resistance?. Mol. Plant-Microbe Inreract..

[19] Ishibashi K., Naito S., Meshi T., Ishikawa M. (2009). An inhibitory interaction between viral and cellular proteins underlies the resistance of tomato to nonadapted tobamoviruses. Proc. Natl. Acad. Sci. USA.

[20] Fujisaki K., Iwahashi F., Kaido M., Okuno T., Mise K. (2009). Genetic analysis of a host determination mechanism of bromoviruses in *Arabidopsis thaliana*. Virus Res..

[21] Sardaru P., Sinausía L., López-González S., Zindovic J., Sánchez F., Ponz F. (2018). The apparent non-host resistance of Ethiopian mustard to a radish-infecting strain of Turnip mosaic virus is largely determined by the C-terminal region of the P3 viral protein. Mol. Plant Pathol..

[22] Shan H., Pasin F., Tzanetakis I.E., Simón-Mateo C., García J.A., Rodamilans B. (2018). Truncation of a P1 leader proteinase facilitates potyvirus replication in a non-permissive host. Mol. Plant Pathol..

[23] Jaubert M., Bhattacharjee S., Mello A.F.S., Perry K.L., Moffett P. (2011). ARGONAUTE2 mediates RNA-silencing antiviral defenses against *Potato virus X* in Arabidopsis. Plant Physiol..

[24] Nieto C., Rodriguez-Moreno L., Rodriguez-Hernandez A.M., Aranda M.A., Truniger V. (2011). *Nicotiana benthamiana* resistance to non-adapted *Melon necrotic spot virus* results from an incompatible interaction between virus RNA and translation initiation factor 4E. Plant J..

[25] Baruah A., Sivalingam P.N., Fatima U., Senthil-Kumar M. (2020). Non-host resistance to plant viruses: What do we know?. Physiol. Mol. Plant Pathol..

[26] Rai A., Sivalingam P.N., Senthil-Kumar M. (2022). A spotlight on non-host resistance to plant viruses. PeerJ.

[27] Halliwell B., Gutteridge J.M.C. (2015). Free Radicals in Biology and Medicine.

[28] Hernández J.A., Gullner G., Clemente-Moreno M.J., Künstler A., Juhász C., Díaz-Vivancos P., Király L. (2016). Oxidative stress and antioxidative responses in plant-virus interactions. Physiol. Mol. Plant Pathol..

[29] Mittler R., Zandalinas S.I., Fichman Y., Van Breusegem F. (2022). Reactive oxygen species signalling in plant stress responses. Nat. Rev. Mol. Cell Biol..

[30] Doke N., Ohashi Y. (1988). Involvement of an O_2_^·−^ generating system in the induction of necrotic lesions on tobacco leaves infected with tobacco mosaic virus. Physiol. Mol. Plant Pathol..

[31] Rossetti S., Bonatti P.M. (2001). In situ histochemical monitoring of ozone- and TMV-induced reactive oxygen species in tobacco leaves. Plant Physiol. Biochem..

[32] Moeder W., Yoshioka K., Klessig D.F. (2005). Involvement of the small GTPase Rac in the defense responses of tobacco to pathogens. Mol. Plant-Microbe Interact..

[33] Király L., Hafez Y.M., Fodor J., Király Z. (2008). Suppression of *Tobacco mosaic virus*-induced hypersensitive-type necrotization in tobacco at high temperature is associated with downregulation of NADPH oxidase and superoxide and stimulation of dehydroascorbate reductase. J. Gen. Virol..

[34] Bacsó R., Hafez Y.M., Király Z., Király L. (2011). Inhibition of virus replication and symptom expression by reactive oxygen species in tobacco infected with *Tobacco mosaic virus*. Acta Phytopathol. Entomol. Hung..

[35] Knip M., Richard M.M.S., Oskam L., van Engelen H.T.D., Aalders T., Takken F.L.W. (2019). Activation of immune receptor Rx1 triggers distinct immune responses culminating in cell death after 4 hours. Mol. Plant Pathol..

[36] Király L., Albert R., Zsemberi O., Schwarczinger I., Hafez Y.M., Künstler A. (2021). Reactive oxygen species contribute to symptomless, extreme resistance to *Potato virus X* in tobacco. Phytopathology.

[37] Desaint H., Aoun N., Deslandes L., Vailleau F., Roux F., Berthomé R. (2021). Fight hard or die trying: When plants face pathogens under heat stress. New Phytol..

[38] Shirdelmoghanloo H., Chen K., Paynter B.H., Angessa T.T., Westcott S., Khan H.A., Hill C.B., Li C. (2022). Grain-filling rate improves physical grain quality in barley under heat stress conditions during the grain-filling period. Front. Plant Sci..

[39] Kolozsváriné Nagy J., Schwarczinger I., Király L., Bacsó R., Ádám A.L., Künstler A. (2022). Near-Isogenic Barley Lines showenhanced Susceptibility to Powdery Mildew Infection Following High-Temperature Stress. Plants.

[40] Schwarzbach E. (2001). Heat induced susceptibility of mlo-barley to powdery mildew (*Blumeria graminis* D.C. f. sp. *hordei* Marchal). Czech J. Genet. Plant Breed..

[41] Barna B., Harrach B.D., Viczián O., Fodor J. (2014). Heat induced susceptibility of barley lines with various types of resistance genes to powdery mildew. Acta Phytopathol. Entomol. Hung..

[42] Fodor J., Nagy J.K., Király L., Mészáros K., Bányai J., Cséplő M.K., Schwarczinger I., Künstler A. (2024). Heat treatments at varying ambient temperatures and durations differentially affect plant defense to *Blumeria hordei* in a resistant and a susceptible *Hordeum vulgare* line. Phytopathology.

[43] Künstler A., Bacsó R., Albert R., Barna B., Király Z., Hafez Y.M., Fodor J., Schwarczinger I., Király L. (2018). Superoxide (O_2_^.−^) accumulation contributes to symptomless (type I) nonhost resistance of plants to biotrophic pathogens. Plant Physiol. Biochem..

[44] Hamilton R.I., Dodds J.A. (1970). Infection of barley by Tobacco mosaic virus in single and mixed infection. Virology.

[45] Dodds J.A., Hamilton R.I. (1972). The influence of Barley Stripe Mosaic Virus on the replication of Tobacco Mosaic Virus in *Hordeum vulgare* L.. Virology.

[46] Király L., Künstler A., Bacsó R., Hafez Y.M., Király Z. (2013). Similarities and differences in plant and animal immune systems—What is inhibiting pathogens?. Acta Phytopathol. Entomol. Hung..

[47] Liu P.-P., Bhattacharjee S., Klessig D.F., Moffett P. (2010). Systemic acquired resistance is induced by R gene-mediated responses independent of cell death. Mol. Plant Pathol..

[48] Gullner G., Juhász C., Németh A., Barna B. (2017). Reactions of tobacco genotypes with different antioxidant capacities to powdery mildew and *Tobacco mosaic virus* infections. Plant Physiol. Biochem..

[49] Künstler A., Király L., Kátay G., Enyedi A.J., Gullner G. (2019). Glutathione can compensate for salicylic acid deficiency in tobacco to maintain resistance to *Tobacco mosaic virus*. Front. Plant Sci..

[50] Watanabe N., Lam E. (2009). Bax inhibitor-1, a conserved cell death suppressor, is a key molecular switch downstream from a variety of biotic and abiotic stress signals in plants. Int. J. Mol. Sci..

[51] Wang X., Tang C., Huang X., Li F., Chen X., Zhang G., Sun Y., Han D., Kang Z. (2012). Wheat BAX inhibitor-1 contributes to wheat resistance to *Puccinia striiformis*. J. Exp. Bot..

[52] Gaguancela O.A., Zuñiga L.P., Arias A.V., Halterman D., Flores F.J., Johansen I.E., Wang A., Yamaji Y., Verchot J. (2016). The IRE1/bZIP60 pathway and Bax inhibitor 1 suppress systemic accumulation of potyviruses and potexviruses in Arabidopsis and *Nicotiana benthamiana* plants. Mol. Plant-Microbe Interact..

[53] Lu P.-P., Yu T.-F., Zheng W.-J., Chen M., Zhou Y.-B., Chen J., Ma Y.-Z., Xi Y.-J., Xu Z.-S. (2018). The wheat Bax Inhibitor-1 protein interacts with an aquaporin TaPIP1 and enhances disease resistance in Arabidopsis. Front. Plant Sci..

[54] Talarczyk A., Krzymowska M., Borucki W., Hennig J. (2002). Effect of yeast CTA1 gene expression on response of tobacco plants to tobacco mosaic virus infection. Plant Physiol..

[55] Bendahmane A., Kanyuka K., Baulcombe D.C. (1999). The Rx gene from potato controls separate virus resistance and cell death responses. Plant Cell.

[56] Baebler Š., Coll A., Gruden K. (2020). Plant molecular responses to Potato virus Y: A continuum of outcomes from sensitivity and tolerance to resistance. Viruses.

[57] Zurbriggen M.D., Carrillo N., Hajirezaei M.-R. (2010). ROS signaling in the hypersensitive response: When, where and what for?. Plant Signal. Behav..

[58] Lukan T., Županič A., Mahkovec Povalej T., Brunkard J.O., Kmetič M., Juteršek M., Baebler Š., Gruden K. (2023). Chloroplast redox state changes mark cell-to-cell signaling in the hypersensitive response. New Phytol..

[59] Farkas G., Király Z., Solymosi F. (1960). Role of oxidative metabolism in the localization of plant viruses. Virology.

[60] Fodor J., Gullner G., Ádám A.L., Barna B., Kőmíves T., Király Z. (1997). Local and systemic responses of antioxidants to tobacco mosaic virus infection and to salicylic acid. Plant Physiol..

[61] Harrach B.D., Fodor J., Pogány M., Preuss J., Barna B. (2008). Antioxidant, ethylene and membrane leakage responses to powdery mildew infection of near-isogenic barley lines with various types of resistance. Eur. J. Plant Pathol..

[62] Gullner G., Tóbiás I., Fodor J., Kőmíves T. (1999). Elevation of glutathione level and activation of glutathione-related enzymes affect virus infection in tobacco. Free Rad. Res..

[63] Höller K., Király L., Künstler A., Müller M., Gullner G., Fattinger M., Zechmann B. (2010). Enhanced glutathione metabolism is correlated with sulfur-induced resistance in Tobacco mosaic virus-infected genetically susceptible *Nicotiana tabacum* plants. Mol. Plant-Microbe Interact..

[64] Király L., Künstler A., Höller K., Fattinger M., Juhász C., Müller M., Gullner G., Zechmann B. (2012). Sulfate supply influences compartment specific glutathione metabolism and confers enhanced resistance to *Tobacco mosaic virus* during a hypersensitive response. Plant Physiol. Biochem..

[65] Zechmann B., Zellnig G., Urbanek-Krajnc A., Müller M. (2007). Artificial elevation of glutathione affects symptom development in ZYMV-infected *Cucurbita pepo* L. plants. Arch. Virol..

[66] De S., Chavez-Calvillo G., Wahlsten M., Makkinen K. (2018). Disruption of the methionine cycle and reduced cellular glutathione levels underlie potex–potyvirus synergism in *Nicotiana benthamiana*. Mol. Plant Pathol..

[67] Gaffney T., Friedrich L., Vernooij B., Negrotto D., Nye G., Uknes S., Ward E., Kessman H., Ryals J. (1993). Requirement of salicylic acid for the induction of systemic acquired resistance. Science.

[68] Künstler A., Füzék K., Schwarczinger I., Nagy J.K., Fodor J., Király L. (2025). Heat shock confers enhanced susceptibility of barley to a necrotrophic pathogen, *Pyrenophora teres* f. *teres*, leading to a more pronounced redox imbalance. Plant Biol..

[69] Schmittgen T.D., Livak K.J. (2008). Analyzing real-time PCR data by the comparative CT method. Nat. Protoc..

[70] Sarkar D., Rovenich H., Jeena G., Nizam S., Tissier A., Balcke G.U., Mahdi L.K., Bonkowski M., Langen G., Zuccaro A. (2019). The inconspicuous gatekeeper: Endophytic *Serendipita vermifera* acts as extended plant protection barrier in the rhizosphere. New Phytol..

[71] Trujillo M., Altschmied L., Schweizer P., Kogel K.H., Hückelhoven R. (2006). Respiratory burst oxidase homologue A of barley contributes to penetration by the powdery mildew fungus *Blumeria graminis* f. sp. *hordei*. J. Exp. Bot..

[72] Hafez Y.M., Király Z. (2003). Role of hydrogen peroxide in symptom expression of barley susceptible and resistant to powdery mildew. Acta Phytopathol. Entomol. Hung..

[73] Ádám A.L., Farkas T., Somlyai G., Hevesi M., Király Z. (1989). Consequence of O_2_^.−^ generation during a bacterially induced hypersensitive reaction in tobacco: Deterioration of membrane lipids. Physiol. Mol. Plant Pathol..

[74] Hückelhoven R., Kogel K.-H. (1998). Tissue-specific superoxide generation at interaction sites in resistant and susceptible near-isogenic barley lines attacked by the powdery mildew fungus (*Erysiphe graminis* fsp. *hordei*). Mol. Plant-Microbe Interact..

[75] Pogány M., von Rad U., Grün S., Dongó A., Pintye A., Simoneau P., Bahnweg G., Kiss L., Barna B., Durner J. (2009). Dual roles of reactive oxygen species and NADPH oxidase RBOHD in an *Arabidopsis-Alternaria* pathosystem. Plant Physiol..

