# Supplementary material for: Suppressing Symptomless Nonhost Resistance of Barley to Tobacco mosaic virus by Short-Term Heat Stress—Role of Superoxide in Resistance
Source: Plants (Basel). 2025 Sep 2;14(17):2736. doi: 10.3390/plants14172736 (PMC12430613; doi:10.3390/plants14172736)
Supplement: Supplementary file 1 [file plants-14-02736-s001.zip › plants-3791589-supplementary.pdf]

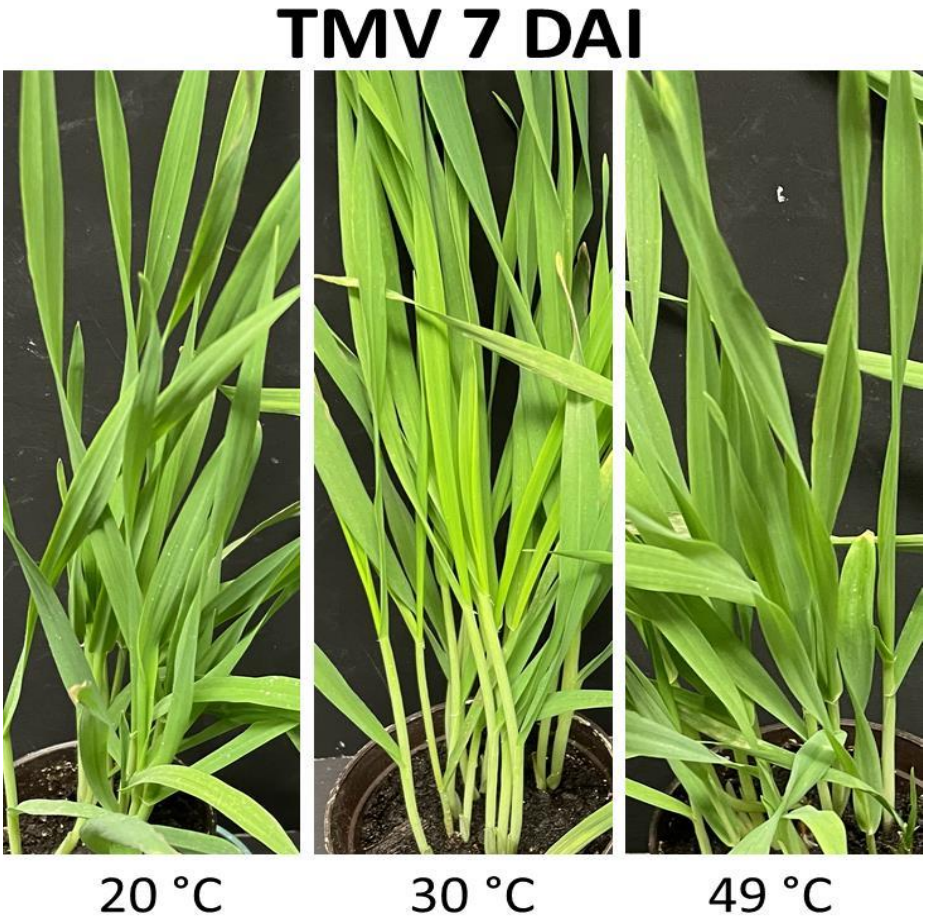

Supplemental Figure S1. Heat stress-compromised nonhost resistance is not associated with any visible symptoms in barley cv. Ingrid plants, at 7 days after *Tobacco mosaic virus* (TMV) inoculation (DAI). Before virus inoculation, barley plants were either constantly kept at 20 °C (20 °C) or pre-exposed to 30 °C for 3 h (30 °C) or to a heat shock (49 °C = 49 °C for 20 s, at 2 h before virus inoculation).
